# Deer Antler Extract Improves Fatigue Effect through Altering the Expression of Genes Related to Muscle Strength in Skeletal Muscle of Mice

**DOI:** 10.1155/2014/540580

**Published:** 2014-02-20

**Authors:** Jaw-Chyun Chen, Chien-Yun Hsiang, Yung-Chang Lin, Tin-Yun Ho

**Affiliations:** ^1^Department of Medicinal Botany and Health Applications, Da-Yeh University, 168 University Road, Dacun, Changhua 51591, Taiwan; ^2^Department of Microbiology, China Medical University, 91 Hsueh-Shih Road, Taichung 40402, Taiwan; ^3^Department of Veterinary Medicine, National Chung Hsing University, 250 Kuo-Kuang Road, Taichung 40227, Taiwan; ^4^Graduate Institute of Chinese Medicine, China Medical University, 91 Hsueh-Shih Road, Taichung 40402, Taiwan

## Abstract

Deer antler is a well-known traditional Chinese medicine used in Asian countries for the tonic and the improvement of aging symptoms. The present study was designed to investigate the antifatigue effect and mechanism of Formosan sambar deer tip antler extract (FSDTAE). The swimming times to exhaustion of mice administered FSDTAE (8.2 mg/day) for 28 days were apparently longer than those of the vehicle-treated mice in forced swim test. However, the indicators of fatigue, such as the reduction in glucose level and the increases in blood urea nitrogen and lactic acid levels, were not significantly inhibited by FSDTAE. Therefore, microarray analysis was further used to examine the anti-fatigue mechanism of FSDTAE. We selected genes with fold changes >2 or <−2 in skeletal muscle for pathway analysis. FSDTAE-affected genes were involved in 9 different signaling pathways, such as GnRH signaling pathway and insulin signaling pathway. All of the significantly expressed genes were classified into 8 different categories by their functions. The most enriched category was muscular system, and 6 upregulated genes, such as troponin I, troponin T1, cysteine and glycine-rich protein 2, myosin heavy polypeptide 7, tropomyosin 2, and myomesin family member 3, were responsible for the development and contraction of muscle. Real-time PCR analysis indicated that FSDTAE increased troponins mRNA expression in skeletal muscle. In conclusion, our findings suggested that FSDTAE might increase the muscle strength through the upregulation of genes responsible for muscle contraction and consequently exhibited the anti-fatigue effect in mice.

## 1. Introduction

Deer antler has been used in traditional Chinese medicines (TCM) and health food about 2000 years. Over the last few decades, commercial deer antler farming has also emerged as a product for the Western dietary supplement market [1]. Deer antler, deer antler base, velvet collagen, and their components, such as mineral elements (Ca, Zn, and Pb), carbohydrates, polypeptides, proteins, and some special cell growth factors [2–5], have been promoted as a treatment to avoid or mitigate the syndromes of diseases including menstrual disorders, arthritis, osteoporosis [6], hypercholesterolemia, and myocardial infarction [7] and to heal chronic wounds [8]. Formosan sambar deer (*Cervus unicolor swinhoei*) and Formosan sika deer (*Cervus nippon taiouanus*) are the indigenous subspecies in Taiwan [9]. Their antler velvet has also been steadily used as TCM and health food more than 400 years in Taiwan. Since 1963, Formosan sambar deer has been started to farming; however, Formosan sika deer populations is extinct in the wild around 1969. Today, more than a half of deer antler source in the Taiwan TCM market is from Formosan sambar deer. In Taiwan, many laborers drink velvet wine for a long time to improve physical strength and reduce fatigue.

Physical fatigue might be caused by energy source depletion and excess metabolite accumulation [10]. The forced-swimming test is one of the commonly used animal models and has been used extensively as a preclinical diagnostic tool for the assessment of antifatigue abilities of Health Food in Taiwan [11]. Research in specific herb(s) or functional supplement(s) is needed to find agents that reduce metabolite accumulation and/or improve energy utilization [12]. Scientists find that the protein extract from sika deer antler base could increase mice swimming time [13, 14]. And Shi et al. indicated that the mechanism of this kind of extracts might be via activating the lactate dehydrogenase activities to reduce the levels of blood lactic acid and also reduce the levels of serum urea nitrogen [15]. Chinese people believe that different regions (tip antler of main beam, middle part of antler, and antler base) of deer antler present different efficacy. And the tip antler is considered the most effective part of velvet. However, there is no study to demonstrate antifatigue activities of tip antler.

In Taiwan, people thought that sambar deer antler is better than sika deer antler. But most deer antler studies focus on the sika deer antler; only a few studies investigate the activities of sambar deer antler [16, 17]. In Taiwan, people speculated that Formosan sambar deer antler may increase endurance exercise performance, but no research confirms such an effect. And antifatigue pathways of deer antler are still unclear. In the present study, we examined the antifatigue effects of Formosan sambar deer tip antler extract (FSDTAE) during a forced-swimming test and evaluated the mechanisms by microarray. Our findings suggested that FSDTAE might increase muscle strength through upregulation of the genes which are responsible for muscle contraction.

## 2. Materials and Methods

### 2.1. Deer Antler Extract Preparation

**Figure 1:**
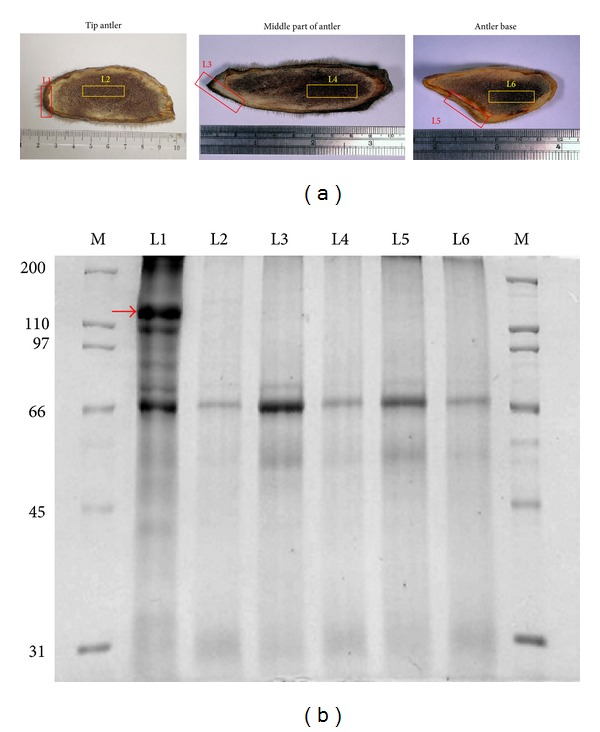
Cross-sections and SDS-PAGE of antler from different layers. (a) Cross-sections of antler from different layers. (b) Proteins of antler were analyzed by electrophoresis on a 10% SDS-PAGE gel. M: marker; L1: Periosteum of tip antler; L2: sponge bone of tip antler; L3: Periosteum of middle part of antler; L4: sponge bone of middle part of antler; L5: Periosteum of antler base; L6: sponge bone of antler base.

Freeze-drying antler of male Formosan sambar deer (Cervus unicolor swinhoei) was provided from the field for deer antler extract sampling. The freeze-drying antlers were divided into three regions, tip antler of main beam, middle part of antler, and antler base (Figure 1(a)). The antler tissues were stored at 4°C until further use. Frozen tissues of antlers were chopped into small pieces and suspended in PBS containing protease inhibitors (PMSF) and homogenized samples were then sonicated briefly and centrifuged at 12000 g for 15 mins at 4°C to remove unbroken tissues and debris. Besides freeze-drying antler tissue, antler correlative products were also collected. These products were also suspended in PBS containing phenylmethanesulfonyl fluoride (PMSF) and sonicated extracts were then centrifuged at 12000 g for 15 mins at 4°C to remove undissolved powder.

### 2.2. Gel Electrophoresis

The total protein concentration of samples was determined by a protein assay kit (Bio-Rad) and compared with quantity standard curve of bovine serum albumin. Protein samples of deer antler were first added SDS sample buffer (final concentrations: 62.5 mM Tris-HCl, pH 6.8, 2% SDS, 10% glycerol, 50 mM dithiothreitol, and 0.1% bromophenol blue), analyzed by 10% sodium dodecyl sulfatepolyacrylamide gel electrophoresis (SDS-PAGE), and stained by Coomassie brilliant blue R-250 stain (Bio-Rad Laboratories).

### 2.3. In-Gel Digestion and Protein Identification by LC/MS/MS

A differentially expressed band of tip antler was excised from the gel, transferred into sterile 2 mL microcentrifuge tubes. Then, this band was destained with 20 mM (NH_4_)HCO_3_ containing 50% methanol in microcentrifuge tubes and dehydrated with 100% acetonitrile. The gel particles were reduced by 10 mM DTT for 45 min and alkylated with 50 mM iodoacetamide for 45 min at RT in 20 mM (NH_4_)HCO_3_ (pH 8.5) solution. Gel particles were washed with 20 mM (NH_4_)HCO_3_ (pH 8.5), dehydrated with acetonitrile, and air-dried. And the proteins were digested by trypsin (Promega, Madison, WI, USA) using an enzyme-to-substrate ratio of 1 : 50 (w/w) for overnight at 37°C. Digested peptides were extracted with 1% trifluoroacetic acid (TFA) in 50% acetonitrile, and samples were dried with a Speed-Vac and redissolved in 0.1% TFA for LC/MS.

Automated nanoliquid chromatography tandem mass spectrometry (nanoLC-MS/MS) was performed for protein analysis. Sample was loaded onto peptide traps and desalted on a precolumn (5 *μ*m, 30 *μ*m I.D. × 5 mm; Dionex, Sunnyvale, CA, USA) after that the peptides were eluted off from the precolumn and separated on an analytical C18 column (3 *μ*m, 15 cm × 75 *μ*m I.D.) connected inline to the mass spectrometer, at 200 nl/min using a linear acetonitrile gradient ranging from 5% to 60% in 0.1% formic acid for a duration of 40 min. For protein identification analysis, the 1s survey scans were acquired over the mass range 400–1600 (m/z) and a maximum of 2 concurrent MS/MS acquisitions. The proteins were identified by searching the LC-MS/MS data of Swiss-Prot database using MASCOT (Matrix Science, UK).

### 2.4. Experimental Animals

Male BABL/c mice (8–10 weeks old) were housed at a room temperature of 25 ± 1°C with a 12/12 h light/dark cycle. Food and water were available ad libitum. They were fed the commercial chow pellet diet during the preliminary period. Those mice were randomly divided into the vehicle and FSDTAE groups (6 mice/group). In all experiments, FSDTAE was dissolved in distilled water and administered orally as a dosage of 8.2 mg per day equal to human dosage. Vehicle groups were fed equal amounts of DDW. All animal procedures were approved by the Institutional Animal Care and Use Committee at the National Chung Hsing University of Taiwan.

### 2.5. Forced Swimming Test

To determine antifatigue activity, the swimming capacity of male Balb/c mice was studied with a 20 cm water pool, and water temperature was maintained at 27°C. Mice were judged to be fatigued when they failed to rise to the water surface to breathe within 8 s period as the index of swimming capacity. On the last day of the preliminary period, the mice that had been deprived of food overnight were subjected to exhaustion, and the swimming capacity was measured. Then the swimming time was estimated as an index of antifatigue effect. At the termination of the study, mice were sacrificed by cervical dislocation. Blood was collected by retroorbital bleeding from mice for the measurement of lactic acid (LA) and blood urea nitrogen (BUN), and serum was obtained by centrifugation at 3000 g for 15 min at 4°C for the measurement of glucose (Glu). Liver and kidney were removed for histological examination.

### 2.6. Total RNA Extraction

Total RNAs were isolated from skeletal muscle tissues around implanted regions with RNeasy Mini kit (Qiagen, Valencia, CA). RNA samples were quantified using the Beckman DU800 spectrophotometer (Beckman Coulter, Fullerton, CA). Samples with A260/A280 ratios more than 1.8 were further evaluated by Agilent 2100 bioanalyzer (Agilent Technologies, Santa Clara, CA). The samples with a RNA integrity number more than 8.0 were accepted for microarray analysis.

### 2.7. Microarray Analysis

Microarray analysis was performed as manufacturer's protocols and described previously [18]. Briefly, RNA was labeled with Cy5 fluorescence (Amersham Pharmacia, Piscataway, NJ) using an Ambion MessageAmp aRNA kit (Ambion, Austin, TX). Labeled samples were mixed and hybridized to the Mouse Whole Genome OneArray (Phalanx Biotech Group, Hsinchu, Taiwan) (*n* = 3) and extracted by an Axon 4000 scanner (Molecular Devices, Sunnyvale, CA). The Cy5 fluorescent intensity of each spot was analyzed by genepix 4.1 software (Molecular Devices, Sunnyvale, CA). The signal intensity of each spot was corrected by built-in control probes signals. We filtered out spots that signal-to-noise ratio was less than 1 or control probes. Spots that passed these criteria were normalized by the limma package of the R program.

### 2.8. Real-Time Polymerase Chain Reaction (PCR) Confirmation of Microarray Results

The expression changes in the 3 genes (troponin T1, troponin I, and tropomyosin 2) of microarray findings were validated using reverse transcription and real-time PCR. Each sample was run in triplicate. TaqMan method was used to amplify and quantitate the transcripts for real-time PCR. TaqMan probes were designed by using corresponding to the GenBank accession numbers for genes in the 3 gene classifier. According to manufacturer's protocols, real-time PCR for each transcript was executed in 96-well fast block optical reaction plates in a reaction volume (25 *μ*L) (containing 1x TaqMan Fast Universal Master Mix, 1x TaqMan gene expression assay, and 2.25 ng of cDNA) using an ABI 7900HT sequence detection system (Applied Biosystems, San Francisco, CA). The cDNA of each gene was amplified under the following cycling conditions: 95°C for 20 seconds, 40 cycles of 95°C for 1 second, and 60°C for 20 seconds. The cycle threshold (Ct) fluorescence values for amplification were recorded for each target gene and *GAPDH* gene. The differences between cycle thresholds for target genes and *GAPDH* in each of the samples were calculated (ΔCt), and the average fold change in expression between FSDTAE-treated and vehicle-treated mice was calculated by the following formula: average fold difference = 2 raised to the power of (ΔCt_*A*_–ΔCt_*V*_).

### 2.9. Statistical Analysis

Data were presented as mean ± standard error. Student's *t*-test was used for comparisons between two experiments. A value of *P* < 0.05 was considered statistically significant.

## 3. Results

### 3.1. Quality Control of Deer Antler by Determining the Expression and Localization of Protein(s) in SDS-PAGE

According to the ancient records of TCM, the efficacies are only significantly changed of the young antler and deer horn, but also significantly different between the parts of tip antler and base. Deer antlers only need 60 days to mature with rapid ossification, then shed in about 5 days. During this procedure, however, the level of various growth factors from deer antlers changes [19]. Polypeptides and proteins were the kinds of the growth factors/active components of deer antler. Many studies also indicated that protein(s) is one of the most active components of deer velvet-related products [20]. In order to control the quality of FSDTAE, we investigated the expression and localization of proteins in periosteum and sponge bone of 3 parts (tip antler, middle part of antler, and antler base) of the deer antler by SDS-PAGE (Figure 1(a)).  Figure 1(b) represents a typical SDS-PAGE pattern of the antler proteome visualized by coomassie blue. The bands derived from periosteum of tip antler with molecular weight at about 110–200 kDa were richer than those derived from other sections of antler. From this gel, the specific band was cut and used to in-gel trypsin digestion and subsequent analysis by LC/MS/MS. Because present public databases contain only a limited entry of deer sequences, we also searched sequences from other vertebrate species (especially mammalian) to find matches. The mass spectrum of the in-gel tryptic digest generated from tip antler is shown in Table 1, and the searching of the peptides led to the identification of 10 different types of collagen-related proteins. Peptides were matched to the theoretical masses with more than 2% coverage of amino acid sequence. Several structure proteins, such as collagen type II alpha 1 and collagen type I alpha 1 (precursor), were the major components in growing tip of antler from red deer (*Cervus elaphus*) [21]. Therefore, we thought that these 10 collagen-related proteins should be one of the major structure proteins in growing tip of Formosan sambar deer antler. Additionally, the content of these 10 collagen-related proteins is determined in each batch of deer antler extract as a quality control by SDS-PAGE.

### 3.2. Effects on Forced-Swimming Test

**Figure 2:**
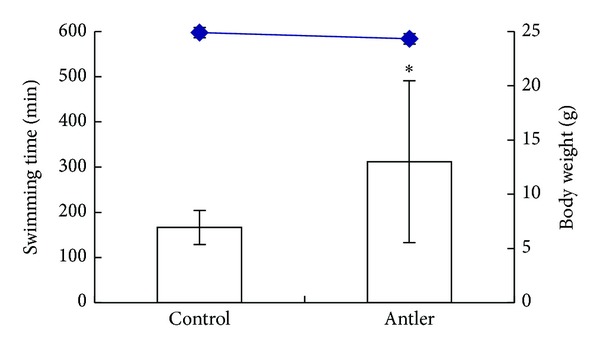
Effects of FSDTAE on swimming time to fatigue in mice. Mice were orally administered FSDTAE or vehicle, and swimming time to fatigue was measured at 28th day. Bars are expressed as an average value in swimming time. Lines are expressed as body weight change. Values are means ± SEM. *P < 0.05, significance between the control and samples.

The antifatigue activity of the FSDTAE (8.2 mg/day) was measured as the exercising endurance capacity of mice using a swimming pool for measuring maximal swimming time. The forced-swimming test was performed at 7th, 14th, 21st, and 28th day to examine the antifatigue effect of FSDTAE. After FSDTAE administration for 7, 14, and 21 days, forced-swimming test present advance effects gradually on swimming duration, but not significantly different to compare with the vehicle group (data not shown). After FSDTAE administration for 28 days, the swimming time of FSDTAE group increased significantly (*P* < 0.05) that compared with the vehicle group (Figure 2). The result indicated that FSDTAE could prolong the swimming time of mice.

### 3.3. Effect of FSDTAE on Fatigue-Related Blood Biochemical Parameters

**Figure 3:**
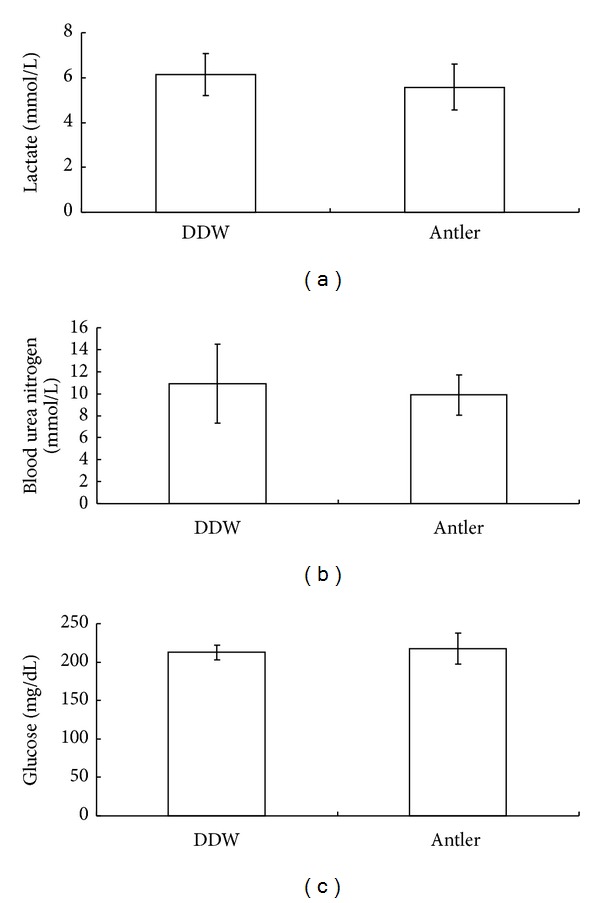
Effects of orally administered FSDTAE on the blood chemical parameters of antifatigue test in mice. Mice were orally administered the FSDTAE or vehicle for 28 consecutive days. Whole blood was collected by retroorbital bleeding from mice for the measurement of lactate (a), BUN (b) and glucose (c). Abbreviations used: BUN, blood urea nitrogen; LAC, lactate; GLU, glucose. Values are means ± SEM.

After forced-swimming tests, the levels of 3 fatigue-related blood biochemical parameters (LA, BUN, and Glu) were determined to clarify the mechanism of FSDTAE (Figure 3). The results indicated slight reductions of LA (Figure 3(a)) and BUN (Figure 3(b)) levels. And only Glu level (Figure 3(c)) was no change in FSDTAE group (*P* > 0.05). Health Food Control Act of Taiwan define that any health food should be safe and noncarcinogenic [11]. Many poisons would change the body weight and food intakes of animals during the experimental period. In the present study, there was no significant difference between the vehicle group and each treatment group (*P* > 0.05) (see Supplementary Figure  1 in Supplementary Materials available online at http://dx.doi.org/10.1155/2014/540580). It showed that orally-administered FSDTAE would not affect appetite and growth in mice. In histological analysis, there were no diverse changes in the appearance of the organs of FSDTAE fed mice compared with vehicle fed mice (see Supplementary Figure  2). The performed experiments revealed that FSDTAE would not cause morphological changes of the tissues of liver and kidney, and there was no tissue damage observed. Results suggested that long-term consumption FSDTAE would not damage liver and kidney.

### 3.4. Microarray Analysis of FSDTAE-Regulated Gene Expression Profile and Pathways of Skeletal Muscle in Mice

When muscle functions are improved, the physiological fatigue will be decreased and the swimming endurance capacity will be increased. Swimming needs several muscles, such as flexor carpi ulnaris, biceps brachii, and triceps brachii that coordinated with each other. Global gene expression profiles in the biceps skeletal muscle of mice given 8.2 mg/day of FSDTAE were obtained using whole-genome oligonucleotide microarrays. The microarray data were analyzed by limma package of bioconductor program to examine the differential expressed genes in the biceps skeletal muscle of mice given with FSDTAE.

To evaluate the antifatigue mechanism of FSDTAE on different pathways, we calculated the average log2 ratios of genes involved in each pathway. Then, we used the “geneSetTest” function to test what biological pathways that FSDTAE treatments could transcriptionally regulate in the biceps skeletal muscle of mice. Here, pathways with FDR <0.05 calculated by “geneSetTest” function followed by multiple testing were considered differentially regulated. Pathway analysis revealed that 9 pathways were regulated (Table 2). Most of the FSDTAE—regulated pathways in the biceps skeletal muscle of mice, were Citrate cycle (TCA cycle) which generates ATP. And two diabetes-related metabolic pathways—insulin signaling pathway and IGF signaling pathway, were regulated. Several systemic hormone pathways, such as prolactin signaling pathway, adipocytokine signaling pathway, serum response factor (SRF) mediated pathway, and GnRH signaling pathway, were regulated. Additionally, the tight junction and adherens junction pathways that play a role in the development of muscle contraction and structure were also regulated. (The genes expressed in each pathway which regulated by FSDTAE in skeletal muscle of mice shown in Supplementary Data Table  1.) These findings suggested that the energy metabolism pathway might not be the major pathway for the antifatigue effects of FSDTAE.

Only 45 genes (28 genes were upregulated and 17 genes were downregulated) were changed more than 2-fold in the biceps skeletal muscle of mice treated with FSDTAE. All of the 45 genes were classified into 11 different categories, such as Muscular System, Energy Metabolism, and Cellular Growth and Proliferation, by their function(s) that annotated through NCBI Gene database (Table 3). Most of enriched category was Muscular System, in which 12 genes such as Myh6 (fold = −7.13), Myh7 (fold = 2.21), HSC70 (fold = −2.82), Tnni3 (fold = −4.85), Actc1 (fold = −2.06), Tpm2 (fold = 2.17), Tnnt1 (fold = 2.29), Tnni1 (fold = 2.38), Myom3 (fold = 4.25), Pln (fold = −2.83), Crip2 (fold = −2.32) and Csrp2 (fold = 2.47), were responsible for the functions of muscle development and contraction. And the second enriched category was Cellular Growth and Proliferation, in which 4 genes such as Spry2 (fold = −3.58), CRBP-II (fold = −2.07), Rarres2 (fold = 2.58) and Pwp2 (fold = 5.15), were reported to be associated with cellular growth and proliferation. These results indicated that the muscle development and contraction should play an important role of antifatigue effects of FSDTAE.

Microarray data also indicated that there were not any carcinogenic genes activated by FSDTAE.

### 3.5. FSDTAE Increases the Levels of Protein Related to Contraction in Skeletal Muscle in Mice

**Figure 4:**
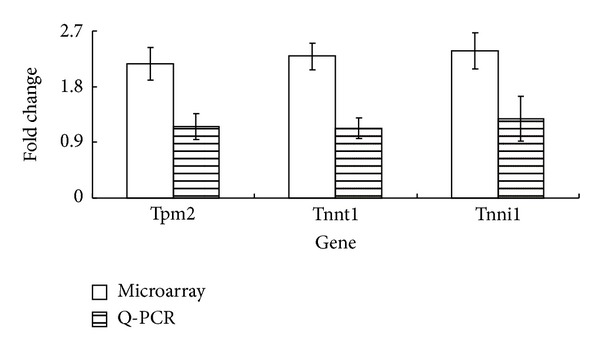
Real-time PCR confirmation of transcript levels of troponins in skeletal muscle samples of FSDTAE-treated and vehicle-treated mice. Average fold elevation of troponins detected in both FSDTAE-treated and vehicle-treated mice skeletal muscle samples by microarray hybridization (white) or real-time RT-PCR (stripe).

Troponins and tropomyosins were contractile and regulatory proteins in skeletal muscle fibers. Changing the expression type of troponins and tropomyosins could improve endurance of muscle. The amounts of troponin T1 (Tnnt1), troponin I (Tnni1), and tropomyosin 2 (Tpm2) were significantly higher in the skeletal muscle of mice treated with FSDTAE relative to mock group mice (Figure 4). FSDTAE significantly increased the levels of these contraction-related proteins in muscle and comparable to that in untreated mice. Integral analysis of these data suggests that FSDTAE could delay the muscle fatigue through modulated expression levels of Tnnt1, Tnni1, and Tpm2.

## 4. Discussion

In mammals, general bony appendages are unable to re-growth again; however, only deer antler can be regenerative annually [22]. Chinese people believe that taking the deer antler could let their organs' function recuperated like a newborn. Chinese people used antlers of various deer species as a conventional therapy for antiaging and rejuvenation. Several of the important growth factors, such as IGF-1, IGF-II, and testosterone, are expressed in the growing deer antler [23], and many people thought that these growth factors lead to the most pharmacological effects of deer antler. However, these growth factors could be found not only in whole deer antler, but also in blood of deer with high level. The 10 collagen-related proteins should be the major structure proteins in growing tip of Formosan sambar deer antler. In Figure 1, we can only find this protein banding in periosteum of tip antler. The result suggests that when the antler tissues start to ossifying this structure protein would be degraded at the same time. Therefore, the 10 collagen-related proteins would more suit as a quality control marker for FSDTAE.

Many TCMs, such as *Cordyceps sinensis* [24], *Panax ginseng* [25], *Panax quinquefolius *[26], *Acanthopanax senticosus *[27], *Antrodia camphorate *[12], and Xiaopi Yishen herbal prescription [28] have been studied as functional foods to improve fatigue effects. Most of these studies investigated the antifatigue function based on exhaustion theory. Therefore, Health Food Control Act of Taiwan defines that fatigue may be related to psychological, physiological, and biochemical factors that are influenced by the mood status of the individual, failure of neuromuscular transmission, and hypoglycemia, and so forth. And the health food with antifatigue effects should not only prolonge the exercise time, but also reduce the levels of several fatigue-related blood biochemical factors, such as GLU, BUN, and LA [11]. According to traditional Chinese medicines theory, deer antler is one of the most active drugs to improve aging and fatigue effects. Our data present that FSDTAE could be prolonged about twice of the forced-swimming time significantly, but weakly reduced the levels of BUN and LA. Although pathway analysis revealed that the Citrate cycle (TCA cycle) is apparently regulated by FSDTAE (Table 2), only one energy metabolism-related gene, Carbonic anhydrase 3 (Car3), is upregulated more than 2-fold. Car3 plays multiple important biological activities such as regulating intracellular pH, and resists some fatigue-related substances [29, 30]. Upregulation Car3 may be the reason to explain that FSDTAE could weakly reduce the levels of BUN and LA. Insulin could increase muscle cells glucose uptake and decrease blood plasma glucose level. FSDTAE could regulate the insulin signaling pathway; therefore, GLU level in FSDTAE group was not significantly higher than control group. So, the exhaustion theory is not very suitable to explain antifatigue effects of FSDTAE. These results suggested that FSDTAE should be able to improve exercise-induced fatigue through different mechanisms.

Every kind of exercise needs regulated muscle, connective tissue, bone, and the nerves that stimulate the muscles. Exercise endurance can be also improved by increasing muscle weight, regulating expression of slow or fast fiber types, and changing neurotransmission to stimulate the muscle contraction. Therefore, Muscular System genes play an important role of exercise-induced fatigue. Myosin is a highly conserved vertebrate protein, where it provides motor function for muscle contraction and needs to interact with actin to originate the force for cellular movements ranging from cytokinesis to muscle contraction. Myosin is a hexameric protein which is composed of two myosin heavy chain (MYH) subunits and two pair of two kinds of light chain subunits. The MYH provides both the motor and filament-forming functions of the intact myosin molecule [31]. The presence of MYH gene family raises the mystery of whether its isoforms are functionally diverse or whether there is functional redundancy [32]. In Table 3, Myh6 expression is downregulated 7.13-fold and Myh7 is upregulated 2.21-fold in skeletal muscle of FSDTAE-treated group compared with vehicle group. Myh6 and Myh7 are expressed in higher mammalian heart, and there are also the major components of the thick filaments of the sarcomere. Developmental analysis shows that Myh7 not only expressed in the adult human heart, but also expressed in skeletal muscle of vertebrates, such as* Xenopus*, and mouse embryos, and the later stages of mouse embryogenesis of smooth muscle tissues [33]. The Cardiac alpha actin 1 (Actc1) plays a role for cardiac muscle and the Y166C and M305L mutants involved in hypertrophic cardiomyopathy [34]. To our best knowledge, there are not any researches indicating that Myh6 and Actc1 are necessary or needed to highly express in skeletal muscle.

Actin-activated myosin II ATPase supplies the power for skeletal muscle to contract. Troponin complex and tropomyosin forms a composite in the thin filament of skeletal muscle which could regulate intracellular Ca^2+^ transient for ATP hydrolysis during actomyosin cross-bridge cycling to generate muscle contraction [35]. The Ca^2+^-binding subunit troponin C (TnC), the actomyosin ATPase inhibiting subunit troponin I (TnI), and the tropomyosin-binding subunit troponin T (TnT) are the members of the troponin complex [36, 37]. Muscle type-specific isoform genes of TnI, TnT, and tropomyosin would be expressed to adapt variety physiological needs and the results (Table 3) present that Tpm2, Tnnt1, and Tnni1 are upregulated 2.17-, 2.29- and 2.38-fold, individually. Tpm2 (beta-tropomyosin) is expressed in both fast and slow twitch fibers. Skeletal muscle diseases, for example, nemaline myopathy, cap disease, and distal arthrogryposis syndromes, are associated with mutations in Tpm2 [38]. This suggests that FSDTAE could increase the muscle strength by upregulated Tpm2 expression. Endurance training has been shown to bring about significant adaptations to the skeletal muscle. In marathon runners, muscle fiber composition ratio of slow-twitch/fast-twitch muscle is increased after endurance training, and much higher than the sprinter. Tnnt1 (skeletal muscle slow-twitch TnT) is the slow skeletal tropomyosin-binding subunit of the troponin complex and plays an essential role in the regulation of striated muscle contraction. Tnni1 (slow skeletal muscle troponin I) is also the component of the troponin complex and key regulatory protein of contractile function in skeletal muscle. The loss of Tnnt1 results in a recessive Amish nemaline myopathy with lethal respiratory failure. Diaphragm muscle exhibited the atrophy and the decreased ratios of slow versus fast isoforms of TnT, TnI, and myosin when the Tnnt1 gene is deficient. The loss of Tnnt1 also results significantly in impair fatigue tolerance, showing faster and more declines of strength with prolong recovery from fatigue [39]. This suggests that FSDTAE might increase fatigue tolerance through upregulated Tnnt1 and Tnni1 expression.

The M-band, a cytoskeletal structure of the sarcomere, cross-links the myosin and titin filaments in the middle to assure proper interactions for muscle contraction. Myomesin could be found in all kinds of mammalian striated muscles (heart and skeletal muscles) inasmuch as M-protein displays a more restricted expression pattern [40–42]. Schoenauer et al. [42] find that Myom3, the structural component of the M-band, presents relatively high levels in the soleus muscle, a powerful muscle for walking, running, and swimming, of adult animals. Myom3 is upregulated 4.25-fold by FSDTAE-treated. This result also supports the fact that FSDTAE increases ratio of slow versus fast skeletal muscle to improve endurance of FST.

## 5. Conclusion

**Figure 5:**
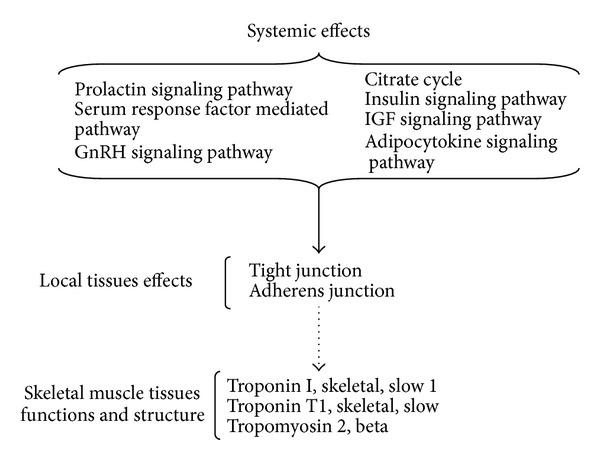
Hypothesis of the mechanism of how deer antler extract to improve the fatigue effects.

In the present study, FSDTAE could increase the swimming time in the forced-swimming test. Microarray results indicated that FSDTAE could regulate 7 pathways to change whole body systemic condition, such as improving energy metabolism. And FSDTAE might regulate functions of local tissues through Tight junction and Adherens junction pathways. Moreover, antifatigue mechanism of FSDTAE might include increase muscle strength through upregulating the genes (Tpm2, Tnnt1, and Tnni1) which are responsible for muscle contraction and increase the ratios of slow versus fast isoform skeletal muscle (Figure 5).

## Supplementary Material

Supplementary Materials: Figure 1 shows that oral administration of FSDTAE for consecutive 28 days had no effect on body weight and food intake. Figure 2 shows that oral administration of FSDTAE for consecutive 28 days displayed no hepatotoxicity and renal toxicity. Table 1 shows the list of genes in each pathway which was regulated by FSDTAE in skeletal muscle of mice.

## Figures and Tables

**Table 1:** LC/MS/MS identification of specific protein from tip antler.

Version number	Protein identification	Coverage (%)	Matched peptide Sequences	Taxonomy
gi∣254221095	Single Type I Collagen Molecule, Rigid Body Refinement	6	GFSGLDGAK; GVVGLIGQR; GFIGADGVAGPK; GVQGPIGPAGPR; GFSGLQGPXGSXGSXGEQGPSGASGPAGPR	*Rattus norvegicus *

gi∣426237753	collagen alpha-1(I) chain	4	GFPGSDGVAGPK; GQAGVMGFPGPK; STGISVPGPMGPSGPR; GETGPAGPAGPIGPVGAR; SGDRGETGPAGPAGPIGPVGAR	*Ovis aries *

gi∣440893297	Collagen alpha-2(I) chain	4	GIPGPVGASGATGAR; HGNRGEPGPAGAVGPAGAVGPR; LPILDIAPLDIGGADQEIR	*Bos grunniens mutus *

gi∣478521049	collagen alpha-1(I) chain isoform 1	3	GFSGLDGAK; GFPGSDGVAGPK; GSPGADGPAGAPGTPGPQGIAGQR	*Ceratotherium simum simum *

gi∣4960163	type I collagen pre-pro-alpha1(I) chain	3	GETGPAGPAGPIGPVGAR; DGEAGAQGPPGPAGPAGER; GQAGVMGFPGPK; GQAGVMGFPGPK	*Canis lupus familiaris *

gi∣524951811	collagen alpha-1(I) chain isoform X1	2	GFSGLDGAK; GAPGADGPAGSPGTPGPQGIAGQR	*Mesocricetus auratus *

gi∣38649122	Collagen, type I, alpha 1	2	GFPGLPGPSGEIGK; GETGPAGVPGPAGPSGPR	*Danio rerio *

gi∣530624898	collagen alpha-1(I) chain	2	GFSGLDGAK; GSPGADGAPGAPGTPGPQGIAGQR	*Chrysemys picta bellii *

gi∣426227338	collagen alpha-2(I) chain	2	TGQPGAVGPAGIR; LPILDIAPLDIGGADQEIR	*Ovis aries *

gi∣343887367	collagen, type I, alpha 2 precursor	2	TGQPGAVGPAGIR; GEPGPAGSVGPAGAVGPR; HGNRGEPGPAGSVGPAGAVGPR	*Sus scrofa *

**Table 2:** Pathway analysis revealed that 9 pathways were regulated by FSDTAE in skeletal muscle of mice.

Pathway	Gene no.	FDR
Citrate cycle	26	3.42*E* − 04
Prolactin signaling pathway	10	5.09*E* − 04
Adipocytokine signaling pathway	69	1.76*E* − 03
Insulin signaling pathway	133	2.14*E* − 03
Tight junction	113	2.35*E* − 03
Serum response factor mediated pathway	10	5.20*E* − 03
GnRH signaling pathway	92	6.25*E* − 03
Adherens junction	73	6.56*E* − 03
IGF signaling pathway	38	1.14*E* − 02

**Table 3:** The expressed genes that were up- or downregulated in the skeletal muscle of FSDTAE-treated group compared with the vehicle group.

Function	Symbol	Gene description	Fold change
Muscular system	Myom3	Mus musculus myomesin family, member 3	4.25
	Csrp2	Cysteine and glycine-rich protein 2	2.47
	Tnni1	troponin I, skeletal, slow 1	2.38
	Tnnt1	troponin T1, skeletal, slow	2.29
	Myh7	myosin, heavy polypeptide 7, cardiac muscle, beta	2.21
	Tpm2	tropomyosin 2, beta	2.17
	Actc1	actin, alpha, cardiac muscle 1	−2.06
	Crip2	Cysteine-rich protein 2	−2.32
	HSC70^a^	Heat shock protein 8	−2.82
	Pln	Phospholamban	−2.83
	Tnni3	troponin I, cardiac 3	−4.85
	Myh6	myosin, heavy polypeptide 6, cardiac muscle, alpha	−7.13

Nervous system development and function	Vps13a	vacuolar protein sorting 13A (chorein)	15.30

Energy metabolism	Car3	Carbonic anhydrase 3	2.14
	Car3	Carbonic anhydrase 3	2.08

Epithelial development and differentiation	Krt10	Keratin, type I cytoskeletal 10	2.75
	Krt10	Keratin, type I cytoskeletal 10	2.35
	Sbsn	suprabasin isoform 1	2.02

Cellular growth and proliferation	Pwp2	Periodic tryptophan protein 2 homolog	5.15
	Rarres2	Retinoic acid receptor responder 2	2.58
	CRBP-II	Cellular retinoic acid-binding protein-II	−2.07
	Spry2	Sprouty homolog 2 (Drosophila)	−3.58

Immune response	C3	Complement component 3	4.07
	C2	Complement component 2	2.45

Miscellaneous genes	Rnu22	Mus musculus small nucleolar RNA	21.85
	Targ3	transforming growth factor alpha regulated gene 3	12.53
	TSEG-2	testis-specific expressed protein 2	12.14
	Tmem165	Transmembrane protein 165	3.54
	Hoxc6	Homeo box C6	3.06
	Eras	ES cell-expressed Ras	2.9
	Olfr1018	Olfactory receptor 1018	2.9
	Serpina1a	Serine peptidase inhibitor, clade A, member 1a	2.34
	Rbp2	Retinol binding protein 2, cellular	−2.07
	Ptgds	Prostaglandin D2 synthase (brain)	−2.11
	Atxn7	ataxin 7	−2.41
	Ube2ql1	ubiquitin-conjugating enzyme E2Q family-like 1	−2.41
	Ankrd13d	Ankyrin repeat domain-containing protein 13D	−2.94
	Magee1	Melanoma antigen, family E, 1	−4.97
	Paip2b	poly(A) binding protein interacting protein 2B	−24.46

Unknown function	D1Ertd622e	hypothetical protein LOC52392	9.87
	5430409L15Rik	RIKEN cDNA 5430409L15 gene	2.81
	1810006K21Rik	Mus musculus RIKEN cDNA 1810006K21 gene	2.3
	A330009N23Rik	RIKEN cDNA A330009N23 gene	−2.13
	Hddc2	HD domain-containing protein 2	−7.28
	A630083H20Rik	RIKEN cDNA A630083H20 gene	−10.18

^a^Other designation Hspa.
