# Supplementary material for: Deer Antler Extract Improves Fatigue Effect through Altering the Expression of Genes Related to Muscle Strength in Skeletal Muscle of Mice
Source: Evid Based Complement Alternat Med. 2014 Feb 20;2014:540580. doi: 10.1155/2014/540580 (PMC3950920; doi:10.1155/2014/540580)
Supplement: Supplementary file 1 — Supplementary Materials: Figure 1 shows that oral administration of FSDTAE for consecutive 28 days had no effect on body weight and food intake. Figure 2 shows that oral administration of FSDTAE for consecutive 28 days displayed no hepatotoxicity and renal toxicity. Table 1 shows the list of genes in each pathway which was regulated by FSDTAE in skeletal muscle of mice. [file 540580.f1.doc]

**Supplementary data**

**Safety evaluation method.** FSDTAE (8.2 mg/day) was administered orally for consecutive 28 days, body weight was recorded before experiment (initial) and after 28 days (final), and food intakes were recorded. After blood sample collecting the mice were sacrificed, the tissues of liver and both kidneys were also collected to prepare slices, then these slices stained with hematoxylin and eosin to examine with microscope for histological analysis.

**FIGURE 1. Effects of orally administered FSDTAE on the body weight and food intake of anti-fatigue test in mice.** Blocks showed the effects of administration of FSDTAE or vehicle on body weight. Body weight changes for FSDTAE (gray; Antler bw) and vehicle (white; DDW bw) groups. Spots showed the effects of administration of pure antler powder on food intake in mice. Food intakes for FSDTAE (round-shaped; Antler fi) and vehicle (square-shaped; DDW fi) groups. Values are means.

**FIGURE 2. Toxic affects analysis of FSDTAE on liver and kidney in mice.** Mice were orally administered with FSDTAE or vehicle for consecutive 28 days. Fatigue was induced by forced-swimming test, and then mice were sacrificed by cervical dislocation. Liver and kidney were removed for histological slices to examine the effect of deer antler. (H&E ×100) (a) Liver; (b) Renal cortex; (c) Renal medulla.

**FIGURE 1**

**FIGURE 2**

(a)


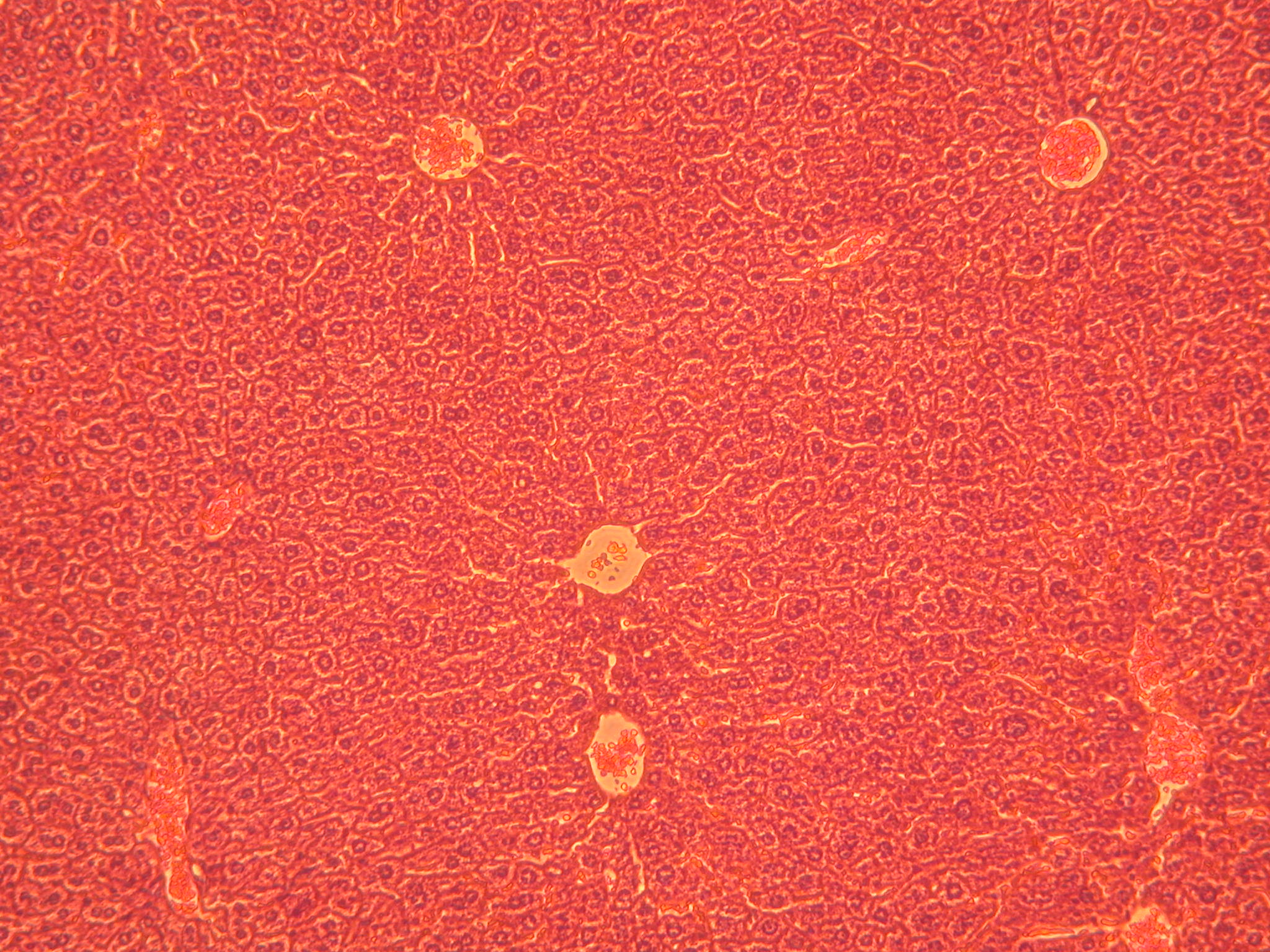

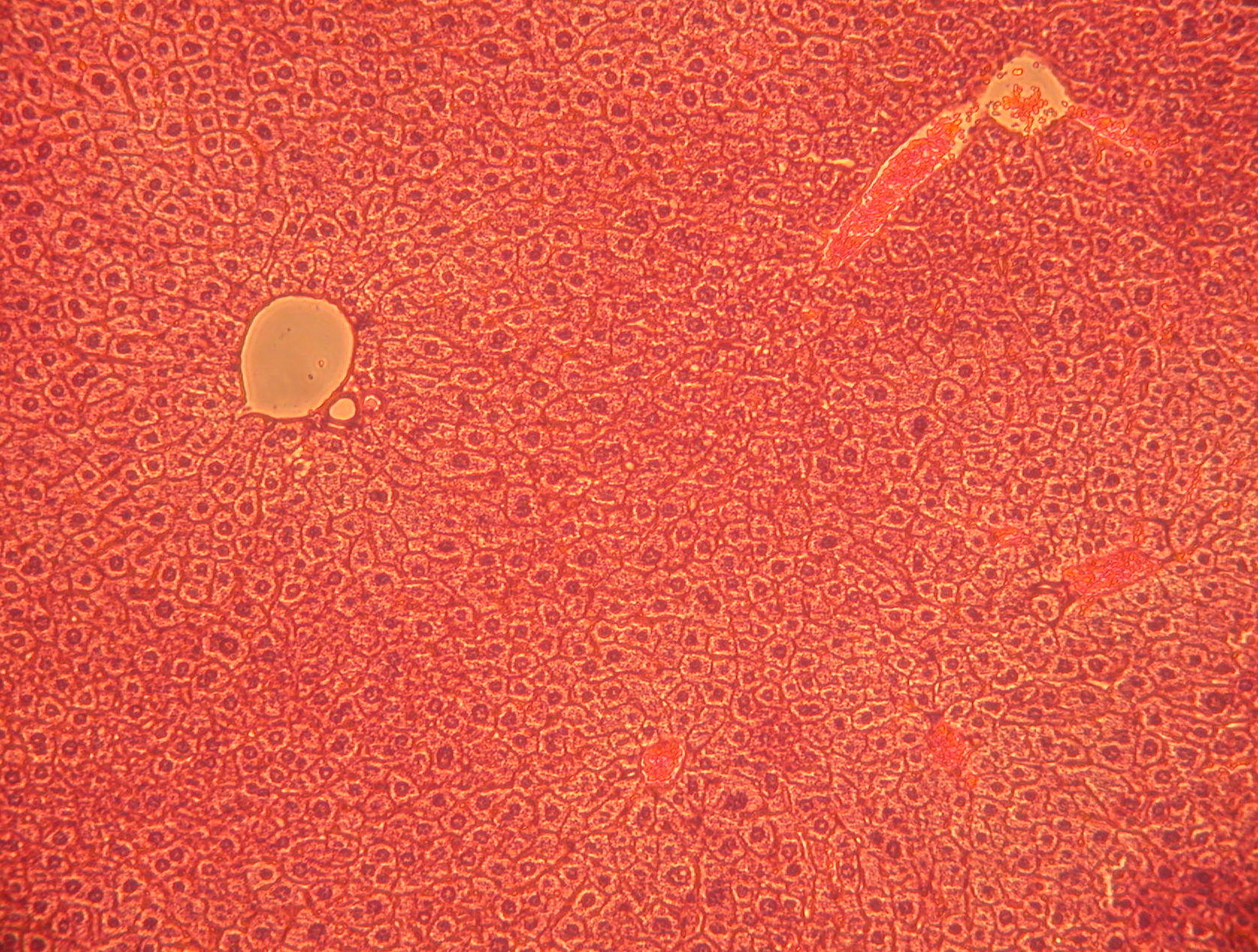


Vehicle group FSDTAE group

(b)


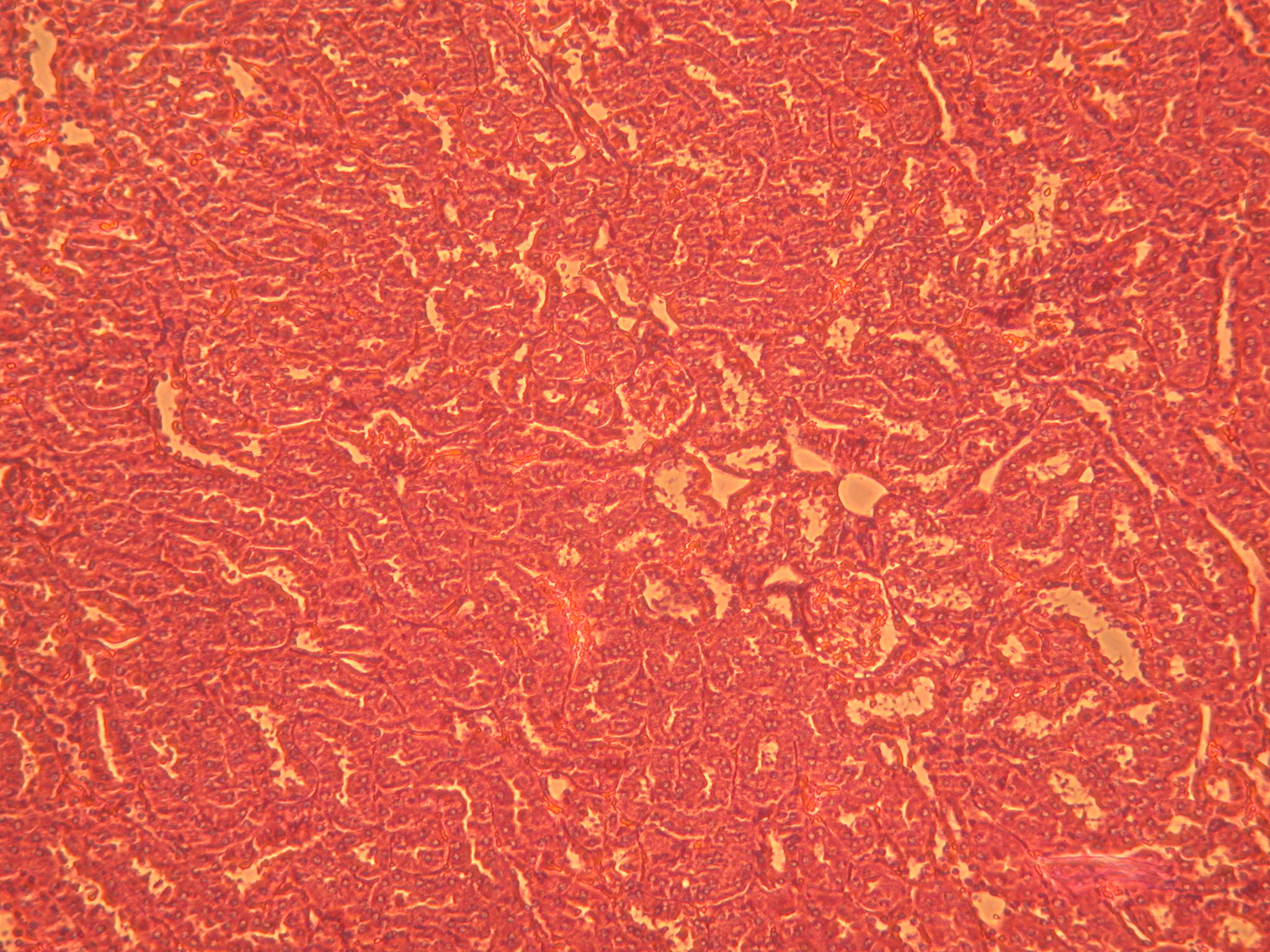

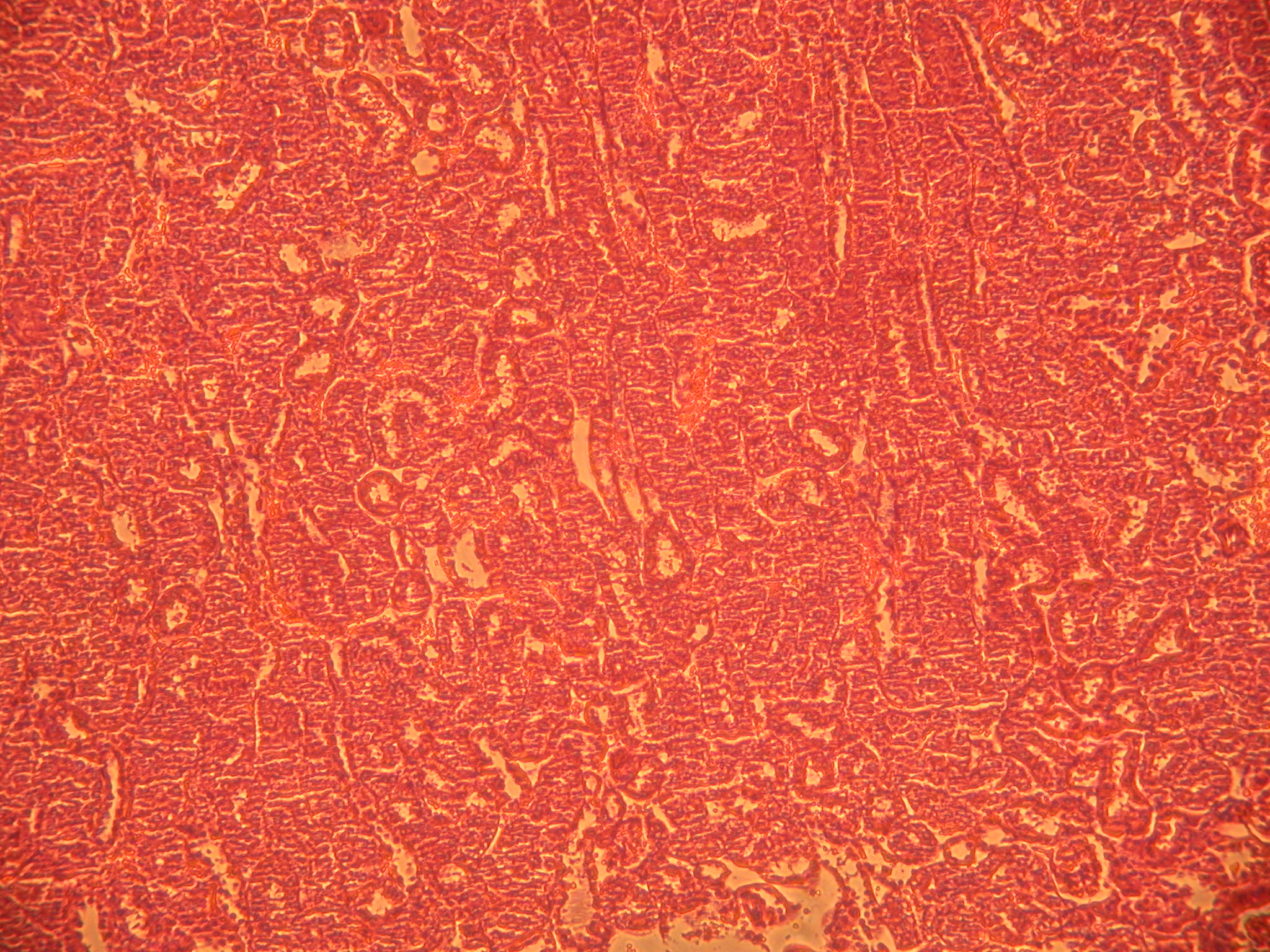


Vehicle group FSDTAE group

(c)


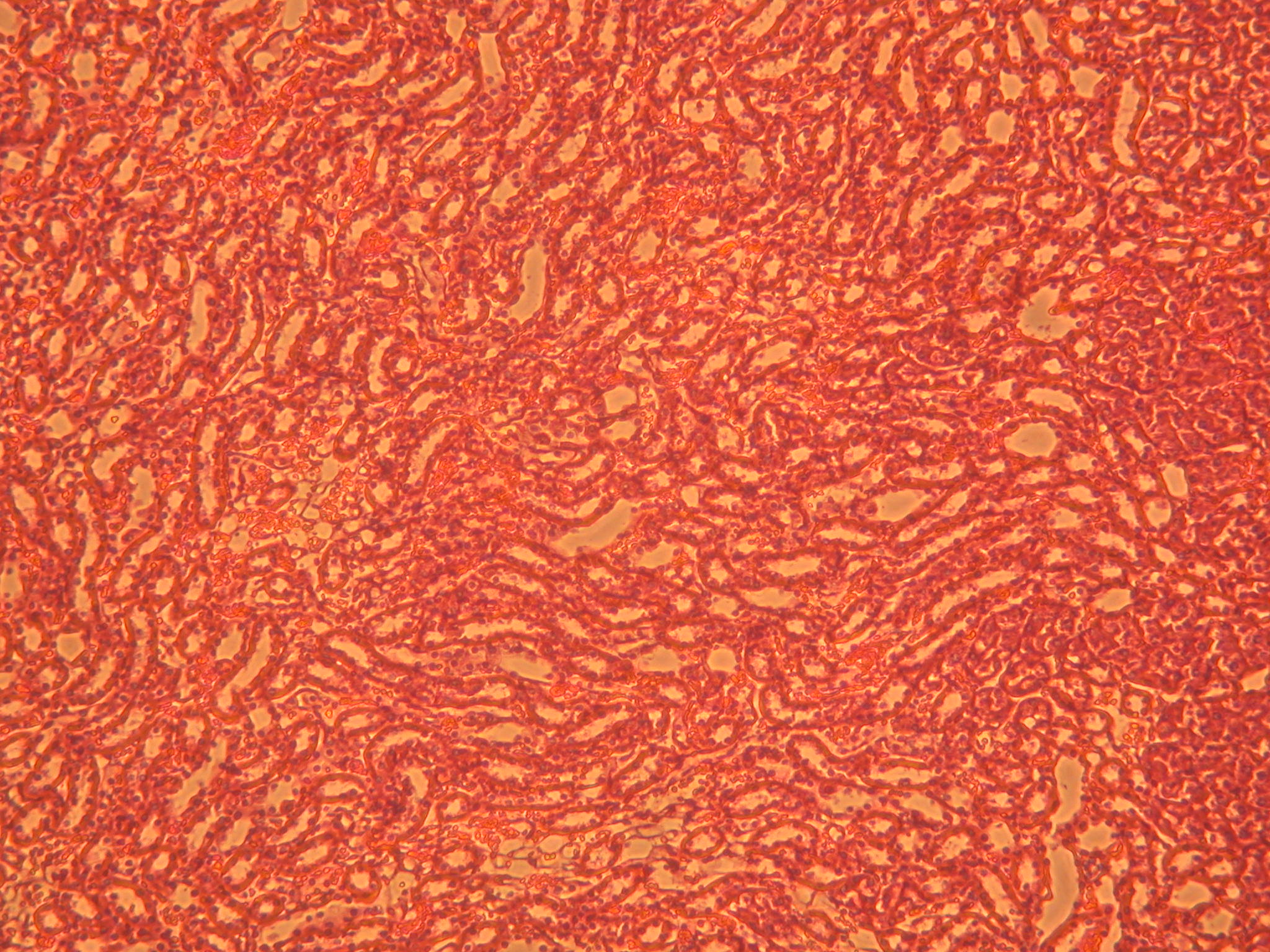

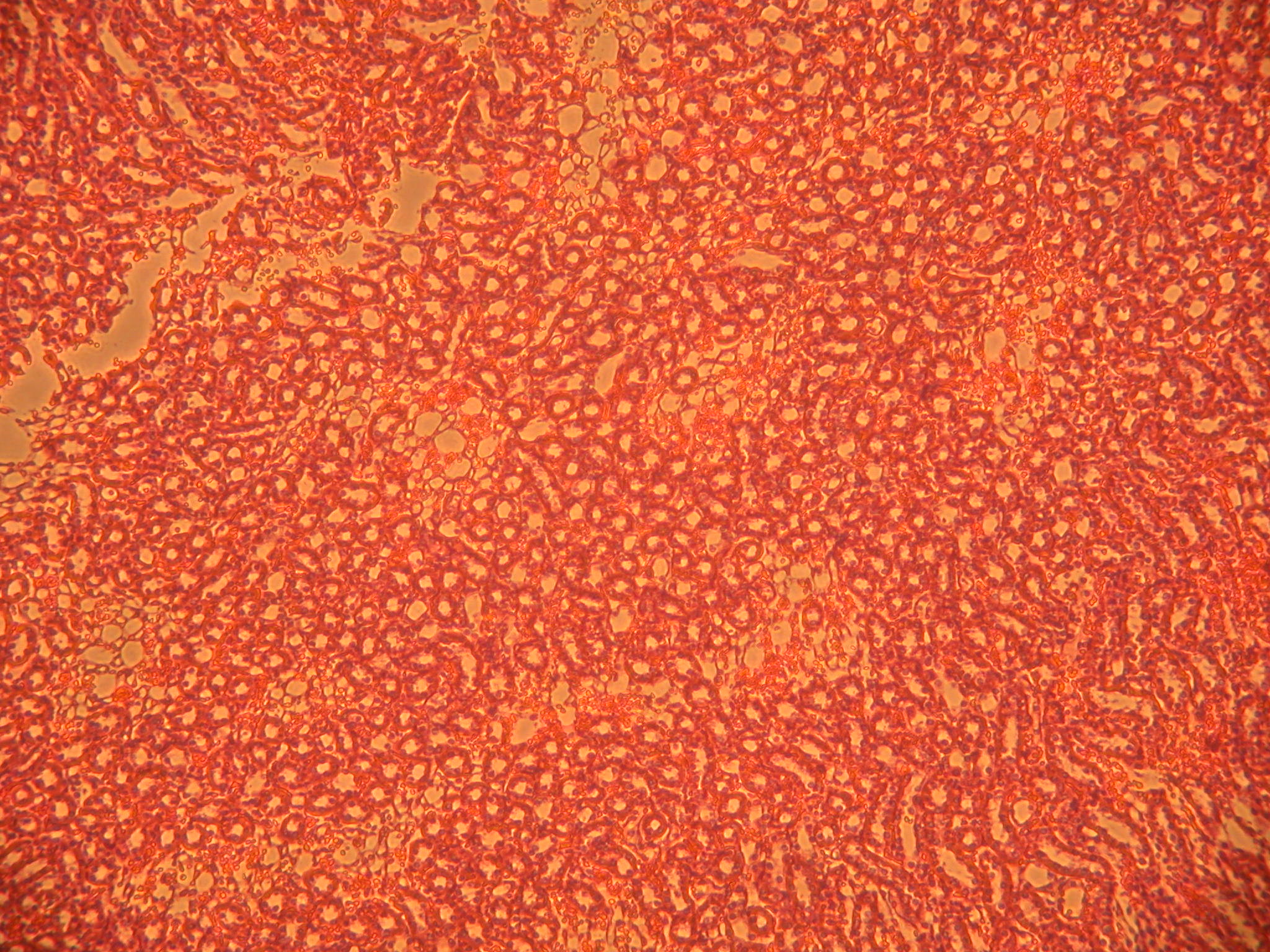


Vehicle group FSDTAE group

**Table 1. Genes expressed in each pathway which regulated by FSDTAE in skeletal muscle of mice.**

| Citrate cycle (TCA cycle) | Gene | Pcx | Idh2 | Idh3g | Fh1 | Idh1 | Acly | Pck2 | Csl | Pck1 | Mdh2 | Sucla2 | Aco1 | Suclg2 | Dlst | Clybl |
| --- | --- | --- | --- | --- | --- | --- | --- | --- | --- | --- | --- | --- | --- | --- | --- | --- |
|  | Gene | Mdh1 | Dld | Sdha | Sdhb | Aco2 | Idh3a | Sdhd | Cs | Sdhc | Ogdh | Suclg1 |  |  |  |  |
| Prolactin Signaling Pathway | Gene | Sfrp2 | Clu | Dcn | Nupr1 | Ctsb | Timp1 | Prl | Bok | Ctsd | Egf |  |  |  |  |  |
| Adipocytokine signaling pathway | Gene | Adipoq | Socs3 | Jak1 | Rxrb | Irs1 | Stat3 | Stk11 | Adipor2 | Cpt1b | Nfkb1 | Slc2a1 | Tnfrsf1a | Camkk1 | Ptpn11 | Irs3 |
|  | Gene | Akt3 | Cd36 | Tradd | Nfkb2 | Cpt1a | G6pc | Traf2 | Prkab1 | Npy | Akt1 | Nfkbib | Pck2 | Pck1 | Camkk2 | Jak3 |
|  | Gene | Prkag2 | Prkaa2 | Cpt1c | Mapk8 | Nfkbie | Jak2 | Rela | Ppargc1a | Acsl3 | Frap1 | Prkaa1 | Irs4 | Tyk2 | Lepr | Agrp |
|  | Gene | Prkag1 | Rxra | Chuk | Mapk10 | Tnfrsf1b | Acsl1 | Acsl4 | Prkcq | Rxrg | Tnf | Acacb | Ikbkb | Akt2 | Ppara | Ikbkg |
|  | Gene | Nfkbia | Cpt2 | Acsl5 | Acsl6 | Slc2a4 | Mapk9 | Prkag3 | Lep | Adipor1 |  |  |  |  |  |  |
| Insulin signaling pathway | Gene | Fasn | Socs3 | Pik3r1 | Phka2 | Socs2 | Cblc | Eif4ebp1 | Mknk2 | Gsk3b | 4932417H02Rik | Lipe | Irs1 | Socs6 | Flot1 | Map2k2 |
|  | Gene | Ppp1r3d | Pkm2 | Pik3r2 | Rps6 | Socs1 | Tsc1 | Mapk3 | Prkar1a | Cblb | Rhoq | Calm4 | Pik3r5 | Crkl | Phkg2 | Prkcz |
|  | Gene | Irs3 | Flot2 | Pklr | Eif4e2 | Akt3 | Pik3ca | Elk1 | Shc3 | Pygl | G6pc | Prkab1 | Kras | Akt1 | Pck2 | Pck1 |
|  | Gene | Pde3b | Pik3cd | Calml3 | Prkar2a | Pik3cb | Nras | Prkaca | Pik3cg | Cbl | Pde3a | Map2k1 | Prkag2 | Ptprf | Pygm | Araf |
|  | Gene | Grb2 | Inpp5d | Prkaa2 | Mapk8 | Rapgef1 | Ppp1r3c | Ppp1r3a | Gys2 | Ptpn1 | Pygb | Calm1 | Prkacb | Fbp1 | Prkar2b | Pik3r3 |
|  | Gene | Rps6kb1 | Prkar1b | Ins2 | Foxo1 | Sos2 | Pfkl | Sh2b2 | Acaca | Ins1 | Ppargc1a | Frap1 | Prkaa1 | Shc2 | Prkci | Irs4 |
|  | Gene | Eif4e | Mknk1 | Prkag1 | Mapk1 | Mapk10 | Bad | Shc1 | Ppp1cc | Sos1 | Srebf1 | Rheb | Tsc2 | Prkx | Crk | Acacb |
|  | Gene | Pfkp | Gck | Exoc7 | Raf1 | Ikbkb | Akt2 | Fbp2 | Gys1 | Pdpk1 | Hras1 | Shc4 | Calm2 | Trip10 | Braf | Rps6kb2 |
|  | Gene | Ppp1ca | Ppp1r3b | Phkg1 | Slc2a4 | Insr | Pfkm | Phka1 | Mapk9 | Prkag3 | Sorbs1 | Calm3 | Phkb | Ppp1cb |  |  |
| Tight junction | Gene | Actn2 | Actn4 | Ash1l | Cldn1 | Llgl2 | Myl6 | Spna2 | Ppp2r3a | Myh9 | Ppp2ca | Tjap1 | Gnai2 | Llgl1 | Vapa | Epb4.1l2 |
|  | Gene | Csda | Tjp1 | Cldn4 | Epb4.1l3 | Mpdz | Cdk4 | Csnk2a2 | Cldn15 | Actn3 | Ppp2r4 | Pard6b | Sympk | Hcls1 | Rras2 | Ppp2r2c |
|  | Gene | Cldn13 | Ppp2r2b | Prkca | Cldn14 | Prkcz | Exoc4 | Mras | Rhoa | Ctnna3 | Cldn10 | Cldn7 | Akt3 | Cldn5 | Prkcb1 | Gnai1 |
|  | Gene | Cldn19 | Myh10 | Rab13 | Csnk2a1 | Prkce | Ppp2r1b | Epb4.1 | Kras | F11r | Akt1 | Myh14 | Cttn | Cldn6 | Cdc42 | Prkcc |
|  | Gene | Cask | Nras | B230120H23Rik | Pten | Ppm1j | Cldn17 | Ocln | Epb4.1l1 | Pard6g | Cldn11 | 4930468A15Rik | Tjp2 | Cldn23 | Cldn16 | Spnb2 |
|  | Gene | 4930412D23Rik | Crb3 | Src | Jam3 | Mpp5 | Exoc3 | Magi1 | Cldn2 | Prkcd | Magi3 | Actg1 | Tjp3 | Ctnna2 | Prkci | Cldn18 |
|  | Gene | Yes1 | Rab3b | Ppp2cb | Cldn9 | Prkcq | Cldn8 | Ppp2r1a | Gnai3 | Rras | Akt2 | Jam2 | Pard3 | Ctnna1 | Prkch | Cldn3 |
|  | Gene | Ctnnb1 | Hras1 | Ppp2r2d | Pard6a | Csnk2b | Inadl | Actb | Actc1 |  |  |  |  |  |  |  |
| Serum response factor Mediated Pathway | Gene | Ptk2 | Rhoa | Vcl | Srf | Egr1 | Tln2 | Fos | Zyx | Tln1 | Actb |  |  |  |  |  |
| GnRH signaling pathway | Gene | Map3k3 | Gnas | Plcb3 | Itpr1 | Map2k2 | Adcy3 | Pla2g4e | Mapk12 | Mapk3 | Pla2g12a | Calm4 | Egfr | Prkca | Adcy2 | Map3k2 |
|  | Gene | Mapk13 | Map2k3 | Elk1 | Prkcb1 | Map3k1 | Mmp14 | Pld2 | Pla2g10 | Pla2g6 | Kras | Cdc42 | Itpr2 | Calml3 | Mapk7 | Cacna1d |
|  | Gene | Cacna1c | Fshb | Nras | Plcb1 | Prkaca | Adcy7 | Map2k6 | Pla2g12b | Map2k1 | Grb2 | Pla2g2e | Pla2g2f | Mapk8 | Map2k7 | Mapk14 |
|  | Gene | Calm1 | Prkacb | Src | Plcb4 | Adcy1 | Prkcd | Gnaq | Pla2g1b | Adcy6 | Sos2 | Mapk11 | Gnrhr | Pla2g4a | Mapk1 | Adcy4 |
|  | Gene | Cacna1f | Mapk10 | Pla2g2c | Cga | Adcy8 | Sos1 | Mmp2 | Camk2b | Camk2a | Camk2d | Prkx | Ptk2b | Map3k4 | Jun | Itpr3 |
|  | Gene | Raf1 | Pla2g5 | Adcy9 | Map2k4 | Hras1 | Calm2 | Pla2g2d | Camk2g | Adcy5 | Pld1 | Gna11 | Hbegf | Mapk9 | Atf4 | Lhb |
|  | Gene | Calm3 | Cacna1s |  |  |  |  |  |  |  |  |  |  |  |  |  |
| Adherens junction | Gene | Actn2 | Actn4 | Farp2 | Smad3 | Tgfbr1 | Baiap2 | Tjp1 | Mapk3 | Csnk2a2 | Actn3 | Igf1r | Egfr | Snai2 | Ptpn6 | Rhoa |
|  | Gene | Ctnna3 | Vcl | Map3k7 | Csnk2a1 | Acvr1c | Lmo7 | Wasf3 | Nlk | Pvrl2 | Cdc42 | Ptprm | Tgfbr2 | Lef1 | Iqgap1 | Ptprf |
|  | Gene | Met | Wasl | Rac2 | Fgfr1 | Acp1 | Wasf1 | Tcf7 | Ptpn1 | Src | Ctnnd1 | Actg1 | Pvrl3 | Ctnna2 | Tcf7l2 | Tcf3 |
|  | Gene | Erbb2 | Yes1 | Crebbp | Was | Ptprj | Mapk1 | Pvrl1 | Smad2 | Fert2 | Ssx2ip | Wasf2 | Fyn | Pvrl4 | Pard3 | Ctnna1 |
|  | Gene | Ctnnb1 | Smad4 | Cdh1 | Acvr1b | Ptprb | Csnk2b | Rac3 | Snai1 | Insr | Actb | Sorbs1 | Rac1 | Actc1 |  |  |
| IGF Signaling Pathway | Gene | Igf1 | Pik3r1 | Igfbp5 | Pcna | Myh1 | Irs1 | Map2k2 | Csk | Nfatc3 | Mapk3 | Parp1 | Cabin1 | Twist1 | Igf1r | Myod1 |
|  | Gene | Mmp13 | Mbp | Bglap1 | Akt1 | Cdkn1b | Map2k1 | Map2k1 | Myog | Grb2 | Map3k5 | Ccnd1 | Mapk14 | Tnfsf11 | Src | Rps6kb1 |
|  | Gene | Spp1 | Mapk1 | Bad | Shc1 | Tnf | Fyn | Raf1 | Vegfa |  |  |  |  |  |  |  |
